# Uplink Non-Orthogonal Multiple Access with Channel Estimation Errors for Internet of Things Applications

**DOI:** 10.3390/s19040912

**Published:** 2019-02-21

**Authors:** Minjoong Rim, Chung G. Kang

**Affiliations:** 1 Dept. of Information and Communication Engineering, Dongguk University, Seoul 04602, Korea; 2 School of Electrical Engineering, Korea University, Seoul 02841, Korea; ccgkang@korea.ac.kr

**Keywords:** IoT, massive IoT, NOMA, random access, channel estimation, QPSK

## Abstract

One of the key requirements for next generation wireless or cellular communication systems is to efficiently support a large number of connections for Internet of Things (IoT) applications, and uplink non-orthogonal multiple access (NOMA) schemes can be used for this purpose. In uplink NOMA systems, pilot symbols, as well as data symbols can be superimposed onto shared resources. The error rate performance can be severely degraded due to channel estimation errors, especially when the number of superimposed packets is large. In this paper, we discuss uplink NOMA schemes with channel estimation errors, assuming that quadrature phase shift keying (QPSK) modulation is used. When pilot signals are superimposed onto the shared resources and a large number of devices perform random accesses concurrently to a single resource of the base station, the channels might not be accurately estimated even in high SNR environments. In this paper, we propose an uplink NOMA scheme, which can alleviate the performance degradation due to channel estimation errors.

## 1. Introduction

Next generation wireless and cellular communication systems are expected to support a variety of services requiring high data rates, low delays, high availabilities, high reliabilities, and large connection densities [1,2,3,4]. Especially, one of the key requirements for the next generation systems is efficiently supporting a huge number of devices for Internet of Things (IoT) applications [4,5,6,7,8,9]. In the future, the prosperity of IoT services can greatly increase the density of devices, which will require massive IoT technologies to support simultaneous random accesses from a large number of devices to a single base station (BS) [4,5,6,7,8,9]. For this purpose, one can use non-orthogonal multiple access (NOMA) schemes, in which signals from devices can be superimposed onto the shared resource and distinguished by spreading or interleaving patterns [10,11,12,13,14,15,16,17]. NOMA schemes can improve the connection density by allowing a greater number of concurrent random accesses compared to other orthogonal schemes. 

Since IoT devices have low transmission power and are often installed in near-shadow areas such as inside-buildings or underground, they typically transmit signals at a very low data rate using repetitions and/or low-rate channel coding. In an uplink NOMA system, data is transmitted at a very low data rate to maintain the required communication coverage, but a large number of users are superimposed on the shared resource, resulting in efficient resource utilization. For low-data-rate transmission, quadrature phase shift keying (QPSK) modulation can be used. QPSK has an advantage over binary phase shift keying (BPSK) in the sense that QPSK allows longer spreading patterns than BPSK, while they have the same bit energy to noise spectral density ratio. In NOMA systems, there is no exact limit on concurrent random accesses, and superimposed signals from many devices can be decoded with the help of interference cancellation techniques. However, preamble or pilot symbols may also be superimposed onto the shared resource to reduce the amount of resources required, and the performance can be degraded due to channel estimation errors, especially when the number of superimposed packets is large [18,19,20,21]. In this paper, we discuss the performance degradation due to channel estimation errors assuming that QPSK modulation is used. We also propose a NOMA scheme robust to channel estimation errors. 

The rest of this paper is organized as follows: Section 2 describes the system model and conventional uplink NOMA scheme. It also addresses the performance degradation due to channel estimation errors. Section 3 proposes a modification to the conventional NOMA scheme to alleviate the performance degradation. Simulation results are shown in Section 4 and conclusions are drawn in Section 5.

## 2. Conventional Uplink NOMA

### 2.1. System Model

In this paper, we consider *K* devices concurrently transmitting packets to a single resource of a BS. The signals from the *K* devices can be distinguished by user-specific pseudo-noise (PN) spreading patterns of length *L*, and we assume a sufficiently large number of spreading patterns so that the collision probability of two or more devices selecting the same spreading pattern is negligible. Let $s_{k}={\left[s_{k,1},\cdots ,s_{k,L}\right]}^{T}$ $\left(|s_{k,l}|=1\right)$ be the spreading pattern vector for the $k^{th}$ device (k=1,⋯,K). If a random-like phase sequence is used for the pattern, in other words, $s_{k,l}=e^{j\phi_{k,l}}$, where $\phi_{k,l}$ is a random variable uniformly distributed from 0 to 2π, then the average correlation value of two different spreading patterns $\rho_{L}^{}\equiv E\{|s_{m}^{H}s_{k}{|}^{2}\}$ (m≠k) can be expressed as follows:

(1) $$\rho_{L}^{\left(random\right)}\equiv E\{|s_{m}^{H}s_{k}{|}^{2}\}=E\left\{{\left|\sum_{l=1}^{L}e^{j(\phi_{k,l}-\phi_{m,l})}\right|}^{2}\right\}=L\;(m\neq k)$$

A spreading pattern can be designed so that the average correlation value can satisfy Equation (1) or the average correlation value can be further reduced by smartly designing the spreading patterns, in other words, $\rho_{L}^{}\leq L$. Hence, we can say that $E\{|s_{m}^{H}s_{k}{|}^{2}\}$$=L^{2}$ for m=k and $E\{|s_{m}^{H}s_{k}{|}^{2}\}=\rho_{L}$≤L for m≠k.

**Figure 1:**
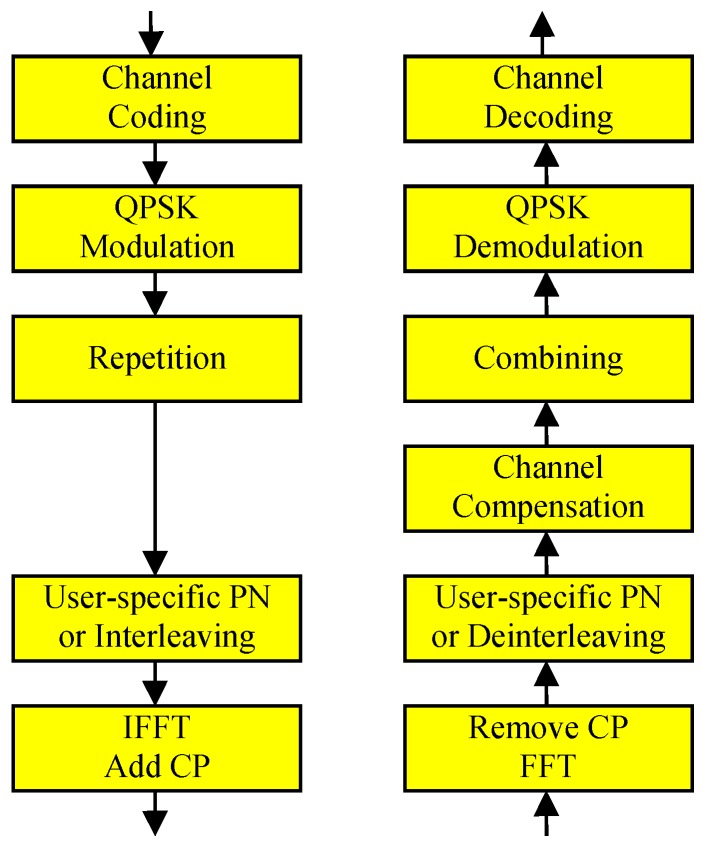
Conventional uplink NOMA.

An uplink NOMA system considered in this paper is assumed to perform the procedure shown in Figure 1. After channel coding and QPSK modulation, the modulated symbols are repeated by *L* times and multiplied by a user-specific PN sequence of length *L*. The spread symbols can be transmitted using narrowband transmission, orthogonal frequency division multiplexing (OFDM), or other transmission scheme. If OFDM is used for the transmission, inverse fast Fourier transform (IFFT) is followed by cyclic prefix (CP) addition. 

Let $x_{k}$ be the QPSK-modulated data signal with $|x_{k}|=1$ for the $k^{th}$ device (1≤k≤K). The transmitted signal vector of the $k^{th}$ device, denoted as $t_{k}$$={\left[t_{k,1},\cdots ,t_{k,L}\right]}^{T}$, can be written as follows:

(2) $$t_{k}=s_{k}x_{k}$$

The BS receives superimposed signals from the K devices, written as

(3) $$r=\sum_{k=1}^{K}\sqrt{P_{k}}e^{j\theta_{k}}t_{k}+n$$

where r is the received signal vector, $P_{k}$ is the received signal power of the $k^{th}$ device, n is the complex noise vector with variance $\sigma^{2}$, and $\theta_{k}$ is the phase of the channel for the $k^{th}$ device, which can be modeled as a random variable uniformly distributed from 0 to 2π. In this paper, we assume a successive interference cancellation technique starting from the device with the strongest received signal power, assuming that $P_{k}$’s can be accurately measured using the pilots at the BS. A successive interference cancellation technique has lower complexity than other complicated reception schemes such as maximal likelihood or parallel interference cancellation techniques. The index for the first decoding device, written as m, is given by:

(4) $$m=\underset{k=1,\cdots ,K}{\arg\max}P_{k}$$

The performance of the first decoded device is important, since it affects the performance of other devices as well. If the decoding process for the first device is failed, the interference cannot be cancelled out from the received signal, and the next device needs to be decoded with lower signal-to-interference-plus-noise ratio (SINR). In order to decode the data from the $k^{th}$ device, the BS performs de-spreading, expressed as follows:

(5) $$\begin{array}{l} s_{m}^{H}r=s_{m}^{H}\left(\sum_{k=1}^{K}\sqrt{P_{k}}e^{j\theta_{k}}t_{k}+n\right)\\ =\sum_{k=1}^{K}\sqrt{P_{k}}e^{j\theta_{k}}s_{m}^{H}s_{k}x_{k}+s_{m}^{H}n\\ =L\sqrt{P_{m}}e^{j\theta_{m}}x_{m}+\sum_{k=1,k\neq m}^{K}\sqrt{P_{k}}s_{m}^{H}s_{k}e^{j\theta_{k}}x_{k}+s_{m}^{H}n \end{array}$$

The first term in Equation (5) includes the signal to be decoded, the second term is the interference from other devices, and the third term is the noise. If perfect channel estimation can be performed, the channel-compensated signal can be written as follows:

(6) $$e^{-j\theta_{m}}s_{m}^{H}r=L\sqrt{P_{m}}x_{m}+\sum_{k=1,k\neq m}^{K}\sqrt{P_{k}}s_{m}^{H}s_{k}e^{j(\theta_{k}-\theta_{m})}x_{k}+e^{-j\theta_{m}}s_{m}^{H}n$$

Considering the QPSK-modulated signal $x_{m}=x_{m,I}+jx_{m,Q}$, the SINR for each real signal, $x_{m,I}$ or $x_{m,Q}$ assuming perfect channel estimation, denoted as $\gamma_{perfect}$, can be written as:

(7) $$\gamma_{perfect}=\frac{L^{2}P_{m}}{\rho_{L}\sum_{k=1,k\neq m}^{K}P_{k}+L\sigma^{2}}$$

and thus the bit error rate (BER) before channel decoding assuming perfect channel estimation, denoted as $p_{perfect}$, can be written as follows:

(8) $$p_{perfect}=Q\left(\sqrt{\frac{L^{2}P_{m}}{\rho_{L}\sum_{k=1,k\neq m}^{K}P_{k}+L\sigma^{2}}}\right)=Q\left(\sqrt{\gamma_{perfect}}\right)$$

### 2.2. Channel Estimation Errors

If pilot signals are also superimposed on the shared resource, the channel estimation performance can be degraded unless large amount of resources are allocated to pilots or the number of concurrently random-accessing devices is small. Assuming that same spreading patterns are used for pilot symbols, the transmitted signal $t_{k}^{\left(p\right)}$ for the pilots of the $k^{th}$ device can be written as:

(9) $$t_{k}^{\left(p\right)}=s_{k}x_{k}^{\left(p\right)}$$

where $x_{k}^{\left(p\right)}$ is the pilot signal with unit magnitude. The BS receives signals from the K devices, expressed as:

(10) $$r^{\left(p\right)}=\sum_{k=1}^{K}\sqrt{P_{k}}e^{j\theta_{k}}t_{k}^{\left(p\right)}+n^{\left(p\right)}$$

where $r^{\left(p\right)}$ is the received signal vector and $n^{\left(p\right)}$ is the corresponding noise with variance $\sigma^{2}$. The BS performs de-spreading for the $m^{th}$ device, written as follows:

(11) $$\begin{array}{l} {\left(x_{m}^{\left(p\right)}\right)}^{*}s_{m}^{H}r^{\left(p\right)}={\left(x_{m}^{\left(p\right)}\right)}^{*}s_{m}^{H}\left(\sum_{k=1}^{K}\sqrt{P_{k}}e^{j\theta_{k}}t_{k}^{\left(p\right)}+n^{\left(p\right)}\right)\\ =\sum_{k=1}^{K}\sqrt{P_{k}}e^{j\theta_{k}}s_{m}^{H}s_{k}{\left(x_{m}^{\left(p\right)}\right)}^{*}x_{k}^{\left(p\right)}+{\left(x_{m}^{\left(p\right)}\right)}^{*}s_{m}^{H}n^{\left(p\right)}\\ =L\sqrt{P_{m}}e^{j\theta_{m}}+\sum_{k=1,k\neq m}^{K}\sqrt{P_{k}}s_{m}^{H}s_{k}e^{j\theta_{k}}{\left(x_{m}^{\left(p\right)}\right)}^{*}x_{k}^{\left(p\right)}+{\left(x_{m}^{\left(p\right)}\right)}^{*}s_{m}^{H}n^{\left(p\right)} \end{array}$$

It may not be easy to achieve satisfactory results with a single pilot symbol, and channel estimate values obtained from multiple repeated pilots can be combined with filtering. The SINR of the channel estimate using N pilot symbols with equal weights of filtering can be expressed as follows:

(12) $$\gamma^{\left(p\right)}=\frac{2NL^{2}P_{m}}{\rho_{L}\sum_{k=1,k\neq m}^{K}P_{k}+L\sigma^{2}}$$

If N is large, then the SINR of the channel estimate can be improved and accurate channel estimates can be obtained at the expense of the waste of resources for pilots. Note that, if N is small and K is large, then the channel estimate might not be accurate even when $P_{m}/\sigma^{2}$ is large, i.e., signal-to-noise ratio (SNR) is large. Unlike orthogonal multiple access transmissions, satisfactory channel estimates may not be obtained with increasing SNR, and it is necessary to consider the effect of channel estimation errors in uplink NOMA systems. Suppose that an inaccurate channel estimate $e^{j(\theta_{m}+\Delta \theta_{m})}$ with phase error $\Delta \theta_{m}$ is used instead of $e^{j\theta_{m}}$ for the $m^{th}$ device. In this case, the channel compensated signal in Equation (6) needs to be rewritten as follows:

(13) $$e^{-j(\theta_{m}+\Delta \theta_{m})}s_{m}^{H}r=L\sqrt{P_{m}}e^{-j\Delta \theta_{m}}x_{m}\;+\sum_{k=1,k\neq m}^{K}\sqrt{P_{k}}s_{m}^{H}s_{k}e^{j(\theta_{k}-\theta_{m}-\Delta \theta_{m})}x_{k}+e^{-j(\theta_{m}+\Delta \theta_{m})}s_{m}^{H}n$$

Due to the channel estimation phase error $\Delta \theta_{m}$ in the range of $|\Delta \theta_{m}|\;<\pi /4$, one of the two real parts in a QPSK symbol is decreased while the other is increased. The SINR values of the two real signals in a QPSK-modulated symbol with the channel estimation phase error $\Delta \theta_{m}$ using the conventional scheme, denoted as $\gamma_{conventional,1}(\Delta \theta_{m})$ and $\gamma_{conventional,2}(\Delta \theta_{m})$, can be written as:

(14) $$\gamma_{conventional,1}(\Delta \theta_{m})=\frac{L^{2}P_{m}}{\rho_{L}\sum_{k=1,k\neq m}^{K}P_{k}+L\sigma^{2}}2\cos^{2}(\pi /4+\Delta \theta_{m})$$

and:

(15) $$\gamma_{conventional,2}(\Delta \theta_{m})=\frac{L^{2}P_{m}}{\rho_{L}\sum_{k=1,k\neq m}^{K}P_{k}+L\sigma^{2}}2\sin^{2}(\pi /4+\Delta \theta_{m}).$$

Hence, the average BER before channel decoding using the conventional scheme, denoted as $p_{conventional}(\Delta \theta_{m})$, can be expressed as follows:

(16) $$\begin{array}{l} p_{conventional}(\Delta \theta_{m})\\ =\frac{1}{2}Q\left(\sqrt{\frac{2L^{2}P_{m}}{\rho_{L}\sum_{k=1,k\neq m}^{K}P_{k}+L\sigma^{2}}}\cos\left(\frac{\pi }{4}+\Delta \theta_{m}\right)\right)+\frac{1}{2}Q\left(\sqrt{\frac{2L^{2}P_{m}}{\rho_{L}\sum_{k=1,k\neq m}^{K}P_{k}+L\sigma^{2}}}\sin\left(\frac{\pi }{4}+\Delta \theta_{m}\right)\right)\\ =\frac{1}{2}Q\left(\sqrt{2\gamma_{\max}}\cos\left(\frac{\pi }{4}+\Delta \theta_{m}\right)\right)\;+\frac{1}{2}Q\left(\sqrt{2\gamma_{\max}}\sin\left(\frac{\pi }{4}+\Delta \theta_{m}\right)\right) \end{array}$$

when $\pi /4\leq \;|\Delta \theta_{m}|\;<\pi /2$, one of the two signals is inverted and Equation (16) still holds. Therefore, Equation (16) can be used for $|\Delta \theta_{m}|\;<\pi /2$.

## 3. Proposed Uplink NOMA

### 3.1. Proposed Sceheme

**Figure 2:**
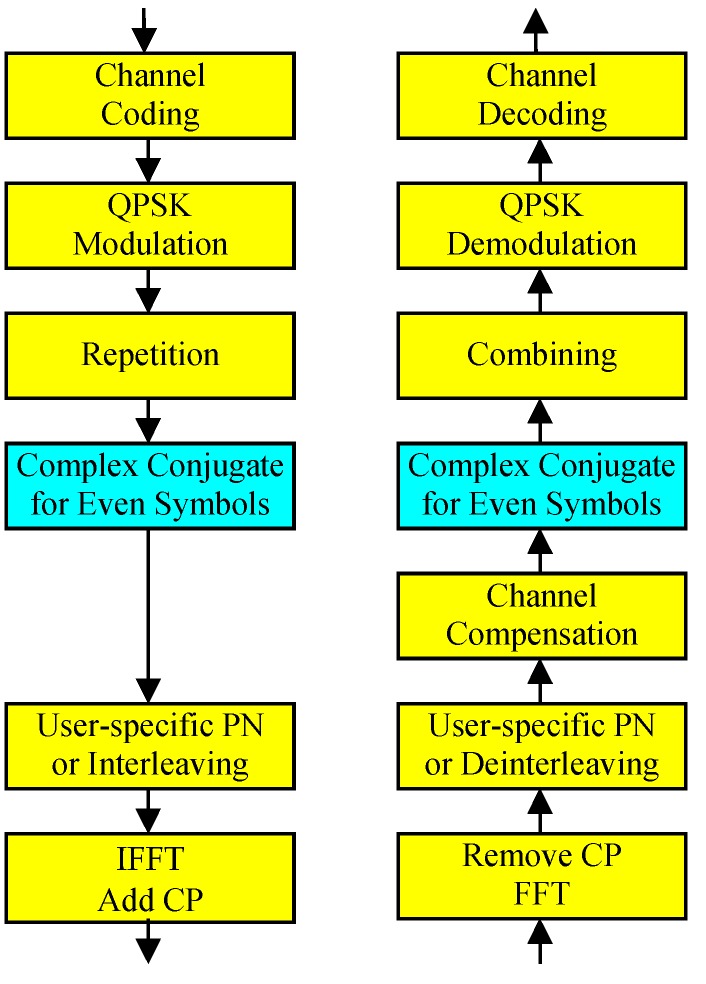
Proposed uplink NOMA.

Unlike orthogonal multiple access systems, channel estimates in uplink NOMA systems might not be accurate even in high SNR environments due to the interference from other devices, and it is desirable to alleviate the performance degradation caused by channel estimation errors. In this paper, we propose an uplink NOMA scheme in which every other symbols after symbol repetition are complex-conjugated before multiplying the spreading pattern, as described in Figure 2. 

Let $I_{even}$ ($I_{odd}$) be the L×L matrix in which the even (odd) diagonal elements are 1 and the others are 0. Notice that $I_{even}+I_{odd}=I$, where I is the L×L identity matrix. In the proposed scheme, the transmitted signal $t_{k}$ for the $k^{th}$ device can be written as follows:

(17) $$t_{k}=I_{odd}s_{k}x_{k}+I_{even}s_{k}x_{k}^{*}$$

If an inaccurate channel estimate $e^{j(\theta_{m}+\Delta \theta_{m})}$ with phase error $\Delta \theta_{m}$ is used instead of $e^{j\theta_{m}}$, the channel compensated symbol can be expressed as:

(18) $$\begin{array}{l} I_{odd}e^{-j(\theta_{m}+\Delta \theta_{m})}s_{m}^{H}r+I_{even}{\left(e^{-j(\theta_{m}+\Delta \theta_{m})}s_{m}^{H}r\right)}^{*}\\ =L\sqrt{P_{m}}x_{m}\cos(\Delta \theta_{m})\\ +\sum_{k=1,k\neq m}^{K}\sqrt{P_{k}}(I_{odd}s_{m}^{H}I_{odd}s_{k}e^{j(\theta_{k}-\theta_{m}-\Delta \theta_{m})}\;+I_{even}s_{m}^{T}I_{even}s_{k}^{*}e^{-j(\theta_{k}-\theta_{m}-\Delta \theta_{m})})x_{k}\\ +(I_{odd}s_{m}^{H}e^{-j(\theta_{m}+\Delta \theta_{m})}+I_{even}s_{m}^{T}e^{j(\theta_{m}+\Delta \theta_{m})})n \end{array}$$

since:

(19) $$I_{odd}s_{m}^{H}I_{odd}s_{m}e^{-j\Delta \theta_{m}}+I_{even}s_{m}^{T}I_{even}s_{m}^{*}e^{j\Delta \theta_{m}}=\frac{L}{2}e^{-j\Delta \theta_{m}}+\frac{L}{2}e^{j\Delta \theta_{m}}=L\cos(\Delta \theta_{m}).$$

Let us define:

(20) $$\rho_{L/2}\equiv E\{|I_{odd}s_{m}^{H}I_{odd}s_{k}{|}^{2}\}=E\{|I_{even}s_{m}^{H}I_{even}s_{k}{|}^{2}\}\;(m\neq k)$$

and:

(21) $$\eta \equiv \frac{\rho_{L}}{2\rho_{L/2}}.$$

In Equation (21), η may depend on the characteristics of the spreading patterns. Note that, if random sequences are used for spreading patterns, then Equation (1) holds and η = 1. From Equation (18), the SINR of each real signal in a QPSK-modulated symbol with the channel estimation phase error $\Delta \theta_{m}$ using the proposed scheme, denoted as $\gamma_{proposed}(\Delta \theta_{m})$, can be written as:

(22) $$\begin{array}{l} \gamma_{proposed}^{}(\Delta \theta_{m})=\frac{L^{2}P_{m}}{2\rho_{L/2}\sum_{k=1,k\neq m}^{K}P_{k}+L\sigma^{2}}\cos^{2}(\Delta \theta_{m})\\ =\frac{L^{2}P_{m}}{\eta \rho_{L}\sum_{k=1,k\neq m}^{K}P_{k}+L\sigma^{2}}\cos^{2}(\Delta \theta_{m}) \end{array}$$

and the BER before channel decoding for the proposed scheme with $\Delta \theta_{m}$, denoted as $p_{proposed}^{}(\Delta \theta_{m})$, can be written as:

(23) $$p_{proposed}^{}(\Delta \theta_{m})=Q\left(\sqrt{\frac{L^{2}P_{m}}{\eta \rho_{L}\sum_{k=1,k\neq m}^{K}P_{k}+L\sigma^{2}}}\cos\left(\Delta \theta_{m}\right)\right).$$

If η is substantially smaller than 1, and channel estimation is perfect, i.e., $\Delta \theta_{m}$ = 0, then:

(24) $$p_{proposed}^{\left(\eta <1\right)}(0)=Q\left(\sqrt{\frac{L^{2}P_{m}}{\eta \rho_{L}\sum_{k=1,k\neq m}^{K}P_{k}+L\sigma^{2}}}\right)\;<p_{perfect}$$

and the proposed scheme does not need to be used. On the other hand, if η is 1 or close to 1, then Equation (23) can be rewritten as:

(25) $$\begin{array}{l} p_{proposed}^{\left(\eta =1\right)}(\Delta \theta_{m})=Q\left(\sqrt{\frac{L^{2}P_{m}}{\rho_{L}\sum_{k=1,k\neq m}^{K}P_{k}+L\sigma^{2}}}\cos\left(\Delta \theta_{m}\right)\right)\\ =Q\left(\sqrt{\gamma_{perfect}}\cos\left(\Delta \theta_{m}\right)\right) \end{array}$$

and the performance can be improved when $\Delta \theta_{m}$ is large, as explained in the next subsection. 

### 3.2. Comparison with Conventional Scheme

With x>0, Q(x) can be expressed as:

(26) $$\begin{array}{l} Q(x)=\frac{1}{\sqrt{2\pi }}\int_{x}^{\infty }\exp\left(-\frac{1}{2}u^{2}\right)du\\ =\frac{1}{2}-\frac{1}{\sqrt{2\pi }}\int_{0}^{x}\exp\left(-\frac{1}{2}u^{2}\right)du \end{array}$$

and thus the second derivative of Q(x) can be written as follows:

(27) $$Q\prime \prime (x)=\frac{1}{\sqrt{2\pi }}x\exp\left(-\frac{1}{2}x^{2}\right)\;>0\;\;(x>0)$$

Since Q(x) is a convex function for x>0 from Equation (27), Equation (16) can be written as:

(28) $$\begin{array}{l} p_{conventional}(\Delta \theta_{m})\\ =\frac{1}{2}Q\left(\sqrt{\gamma_{perfect}}\left(\cos(\Delta \theta_{m})-\sin(\Delta \theta_{m})\right)\right)+\frac{1}{2}Q\left(\sqrt{\gamma_{perfect}}\left(\cos(\Delta \theta_{m})+\sin(\Delta \theta_{m})\right)\right)\\ \;\geq Q\left(\sqrt{\gamma_{perfect}}\cos(\Delta \theta_{m})\right)\;=P_{proposed}^{\left(\eta =1\right)}(\Delta \theta_{m}) \end{array}$$

by the Jensen Inequality [22]. When the channel estimation phase error $\Delta \theta_{m}$ is 0 or π/2, $p_{proposed}^{\left(\eta =1\right)}(\Delta \theta_{m})$ is the same as $p_{conventional}(\Delta \theta_{m})$. But for $0<\Delta \theta_{m}<\pi /2$, the proposed scheme can achieve less BER than the conventional scheme, meaning that the proposed scheme is more robust to channel estimation errors. When multiple packets are superimposed onto the single resource, there can be severe channel estimation errors due to the interference among the devices. In this case, the proposed NOMA scheme can reduce the BER before channel decoding especially when random-like spreading patterns are used and thus η is 1 or close to 1. On the other hand, if the complex-conjugate operations significantly change the characteristics of spreading patterns and η becomes considerably smaller than 1, then there are two adversary effects and it is not easy to predict which is better.

## 4. Simulation Results

In this section, we compare the conventional and the proposed schemes in terms of BER before channel decoding and frame error rate (FER) after channel decoding. Each device transmits 20-byte data (160 bits) with 1/3-rate channel coding (convolutional coding with constraint length 7 and generator polynomial 171, 165, and 133), QPSK modulation, and eight times spreading with random phase sequences. We assume flat fading and consider channel estimation based on eight (N=1), 16 (N=2), and 32 (N=4) resource elements for pilots in addition to ideal channel estimation. Open-loop power control of the transmitting devices may not be perfect and the received signal power is generated uniformly from -3dB to +3dB compared to the operating SNR. Successive interference cancellation is used in the decreasing order of the received power strengths. 

**Figure 3:**
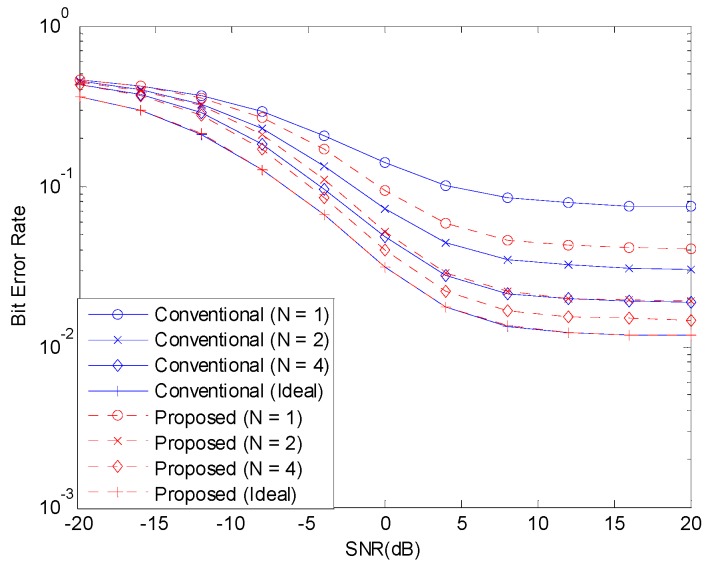
BER before channel decoding with 12 active devices.

**Figure 4:**
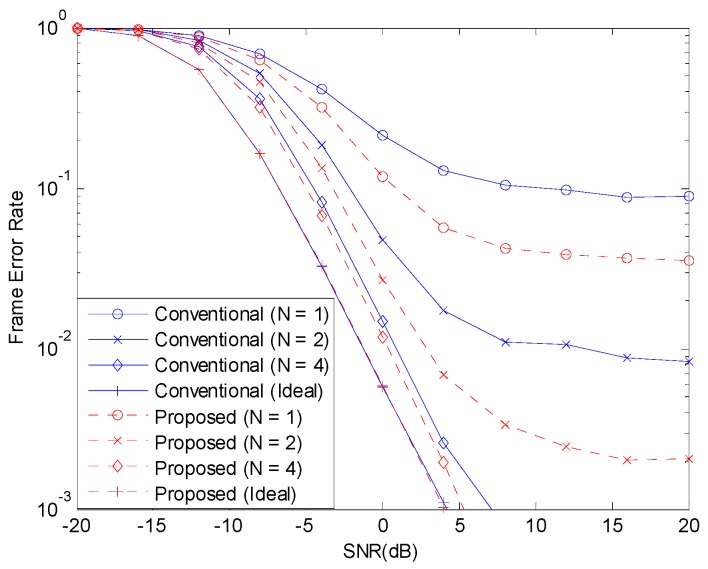
FER with 12 active devices.

Figure 3 and Figure 4 show the BER before channel decoding and the FER performances, respectively, with 12 concurrent accesses to the shared resource. The number of pilot symbols N increases the waste of resource and the accuracy of channel estimation. If the channel estimate is perfect, that is, if N is infinite, the NOMA system can achieve good performance. But, when N is small, the channel estimate is inaccurate and the performance of NOMA system degrades. When the channel estimation is perfect, there is no difference in the performances of the two schemes. With channel estimation errors, the performance is degraded but the performance degradation can be alleviated with the proposed scheme. 

**Figure 5:**
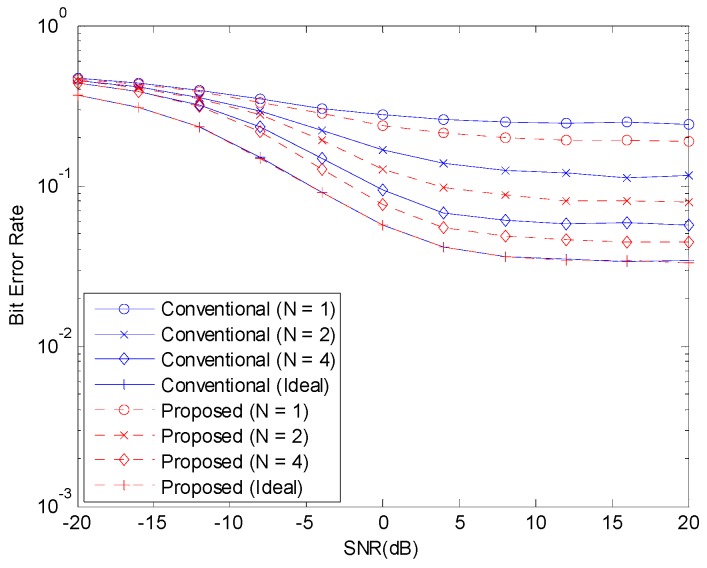
BER before channel decoding with 20 active devices.

**Figure 6:**
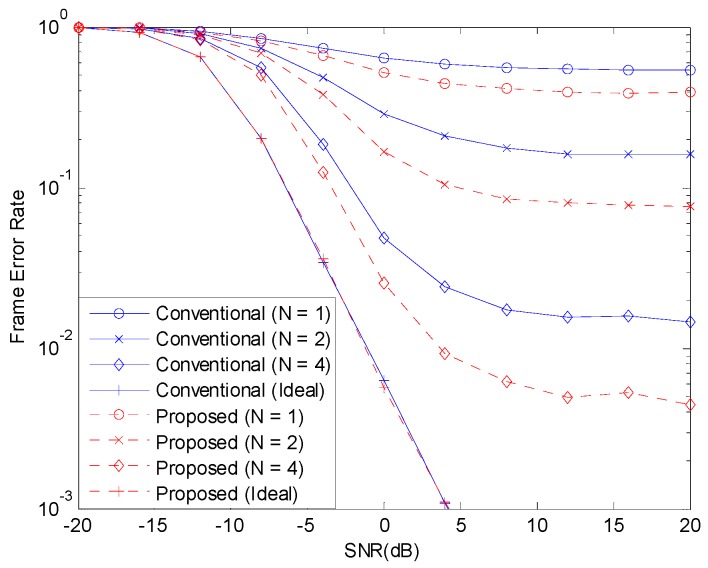
FER with 20 active devices.

Figure 5 and Figure 6 show the BER before channel decoding and the FER performances, respectively with 20 active devices. As the number of active devices increases, the inaccuracy of the channel estimates increases and it is important to consider the effect of channel estimation errors.

## 5. Conclusions

As IoT services become richer, there is a growing demand for massive connectivity technologies to support simultaneous accesses from a large number of devices to a single BS. In order to fulfill the requirement, one can use NOMA schemes, which improve the connection density by allowing signals from multiple devices superimposed onto the shared resource. In this paper, we discussed the issues relating to performance degradation due to channel estimation errors in uplink NOMA systems. When pilot signals are superimposed onto the shared resource as well, and a large number of devices perform random accesses concurrently, the channels might not be accurately estimated even in high SNR environments. This paper proposed an uplink NOMA scheme, which can alleviate the performance degradation due to channel estimation errors. An optimized scheme assuming perfect channel estimation might not be the best with inaccurate channel estimates and channel estimation errors need to be considered for uplink NOMA schemes in order to support a large number of concurrent random accesses. There are a large number of variations in uplink NOMA systems and more rigorous theoretical analysis needs to be performed with diverse uplink NOMA systems in the future.
